# Editorial: Non-genetic adaptive drug resistance in cancer

**DOI:** 10.3389/fcell.2022.998339

**Published:** 2022-11-18

**Authors:** Michaël Cerezo, Xiaoxiao Sun, Shensi Shen

**Affiliations:** ^1^ INSERM U1065 Equipe 12, Centre Méditerranéen de Médecine Moléculaire (C3M), Université Côte d’Azur, Nice, France; ^2^ Department of Pharmaceutical Chemistry, University of California, San Francisco, San Francisco, CA, United States; ^3^ Department of Thoracic Surgery, Institute of Thoracic Oncology, West China Hospital, Sichuan University, Chengdu, China

**Keywords:** cellular plasticity, drug resistance, persistent cancer cells, therapeutic targets, tumor relapse, non-genetic adaptation

One of the main characteristics of cancer cells is their incredible plasticity, which is possibly the origin of tumor resistance to diverse therapeutics. During cancer treatment and in particular with targeted therapies, tumor cell evolution can be divided into three phases. First, the tumor shrinking phase characterized by the dramatic tumor mass decrease. Second, the residual disease phase during which persistent cancer cells are mostly “dormant,” and tumor mass remains undetectable at the radiological level. Third, the tumor escaping phase in which the reprogrammed genetic features of residual cancer cells cause an explosive tumor progression. One of the major questions regarding this stepwise evolution process is whether the surviving malignant cells are reprogrammed and/or selected during drug treatment, thus serving as a reservoir for the subsequent resistance and eventual tumor relapse. With the great advances in tumor resistance research during the last decade, non-genetic adaptive resistance mechanisms emerge as a quintessential component of cancer evolution. Multiple adaptive traits involved in drug resistance have been revealed, such as the capacity for tumor cells to alter their epigenetic states, rewire the intracellular signaling network, or co-opt extrinsic cell-to-cell symbiosis. In this Research Topic collection, we present the state-of-art in non-genetic adaptive drug resistance in cancer and cover topics that include tumor heterogeneity, accessible chromatin rearrangement in resistant cancer cells, mechanisms of drug efflux, gene rearrangement participating in cancer resistance, and important mechanisms that regulate genomic repeat elements in cancer.

Rabé et al., explored the heterogeneity of a glioblastoma cell line under temozolomide treatment. Using an elegant multicolor barcoding strategy, the authors demonstrated that the resistance to temozolomide does not result from clonal selection, but rather that dynamical persistence is induced by the temozolomide treatment *per se*. They identified a four-gene signature encoding for proteins with extracellular activities that could be implicated in remodeling the microenvironment, thus inducing the survival and the regrowth of resistant cells.

At the epigenetic level, Wang et al. explored doxorubicin dependent non-genetic resistance in breast cancer. They showed that chromatin accessibility in resistant cancer cells dictates a specific gene expression landscape, in which putative therapeutic targets could be further investigated to improve therapeutic strategies.

Nicholson et al. identified PKCϵ as an important player of cancer cell aggressiveness and resistance in Acute Myeloid Leukemia (AML). PKCϵ was involved in the upregulation of the efflux pump P-GP, leading to a poor clinical prognosis. In consequence, their work highlights the importance of the drug efflux pump in cancer cell adaptation to treatment.

In a case report, Feng et al. identified a novel serine/threonine kinase 3 (STK3)-ALK rearrangement in a patient with advanced non-small cell lung cancer (NSCLC). Interestingly, this patient was resistant to the ALK inhibitor crizotinib but responsive to alectinib, a different drug that also inhibits ALK. This work underscores the potential benefit of large-scale, unbiased approaches (i.e., next generation sequencing) in dissecting cancer plasticity and proposing alternative strategies to overcome drug resistance.

Kermi et al. offered a systematic overview of the role of genomic repeat elements including retrotransposons in cancer aggressiveness and resistance to therapies. Their work opens a new route toward the exploitation of transposable elements that could be the “Achilles’ heel” of tumor cells, especially in the context of immunotherapy treatments. In fact, recent studies have shown that dysregulation of transposable elements could potentiate cancer cell immunogenicity by increasing neoantigen presentations.

Taken together, this Research Topic collection shows the multilevel complexity of non-genetic adaptive resistance in cancer treatment and offers enthusiastic prospects in this domain. With the advent of novel single cell analysis technologies and lineage tracing, the underlying biological mechanisms of non-genetic tumor resistance will be further explored in a higher resolution. Future research will be further conducted in topics like drug resistance-associated immunosurveillance, adaptive genetic instability, cell invasiveness, and multicellular symbiosis.

We would like to thank all the authors, co-authors, and reviewers who accepted our invitation and contributed to this Research Topic, and we sincerely hope that the data and information conveyed will be beneficial to the cancer research community.

